# Supplementary material for: Gibberellin transport affects lateral root growth through HY5 in response to far-red light
Source: Plant Cell. 2025 Aug 16;37(9):koaf200. doi: 10.1093/plcell/koaf200 (PMC12448942; doi:10.1093/plcell/koaf200)
Supplement: koaf200_Supplementary_Data [file koaf200_supplementary_data.zip › Supplementary Data.pdf]

Gibberellin transport affects (lateral) root growth through HY5 during Far-Red light enrichment.

Kasper van Gelderen[1,3#], Kyra van der Velde[1,2], Chia-kai Kang[1], Jessy Hollander[1], Alicia Koppenol[1], Orfeas Petropoulos[1,5], Putri Prasetyaningrum[3,4], Tuğba Akyüz[1] and Ronald Pierik[1,2#].

Affiliation:
1: Plant-Environment Signaling, Dept. of Biology, Utrecht University, Padualaan 8 3584CH, Utrecht, The Netherlands.
2: Laboratory of Molecular Biology, Wageningen University and Research, Droevendaalsesteeg 1, 6708PB, Wageningen, The Netherlands.
3: Light Signaling and Cell Biology, Centre for Organismal Studies, Heidelberg University, Im Neuenheimer Feld 230, 69120, Heidelberg, Germany.
4: Current address: Department of Molecular Plant Physiology, Faculty of Biology, University of Freiburg, Schänzlestrasse 1, 79104 Freiburg i. Br., Germany.
5: Current address: RADICLE crops Wageningen
# corresponding authors

Supplemental figures

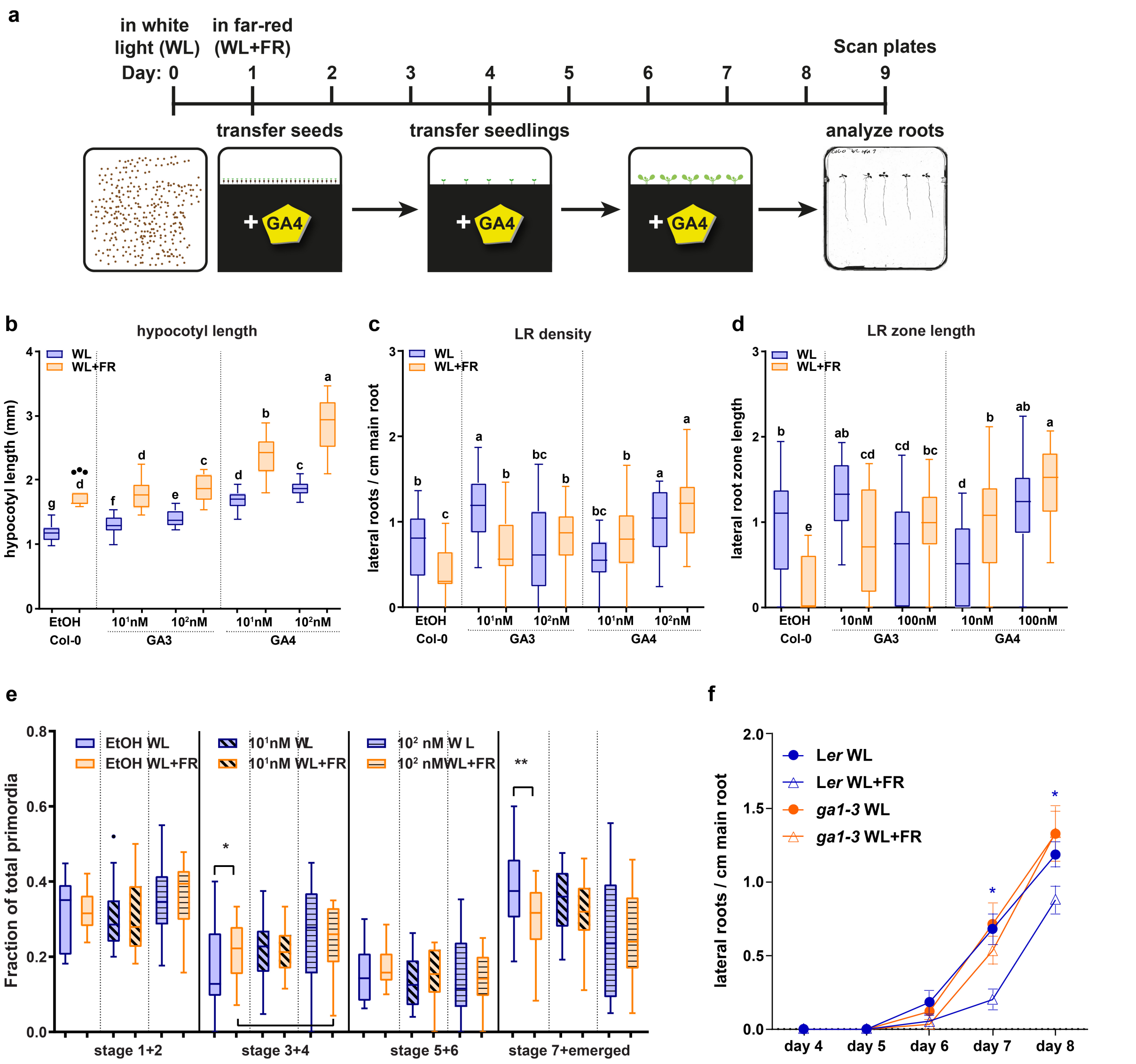

Supplemental Figure S1 | Gibberellins are involved in FR-shoot-induced changes in root development. Supports Figure 1 (a) Growth scheme for phenotyping experiments of Figures 1,2,6,10 and S1, S4, S6. (b-d) Seedlings were grown for 9 days according to the schedule on (a). Scans of 8d old seedlings were analyzed on (b) hypocotyl length; (c) lateral root density; (d) lateral root zone length. (e) A similar experiment to Figure 1 was performed with GA4 addition, however seedlings were fixed afterwards and analyzed with DIC microscopy on lateral root primordia stages. (f) Lateral root density of experiment performed similarly to the schedule in (a), with the addition that seeds were surface sterilized with ethanol and bleach and then taken up in 0.1% agarose, which for the *ga1-3* mutant contained 10^2 nM of GA4, in order to start germination of this otherwise non-germinating line. Means were statistically significant based on a 2-way ANOVA; letters denote significant difference between treatments based on a post-hoc tukey test ($p<0.05$, $n > 20 < 30$). Boxplot whiskers show the distribution.

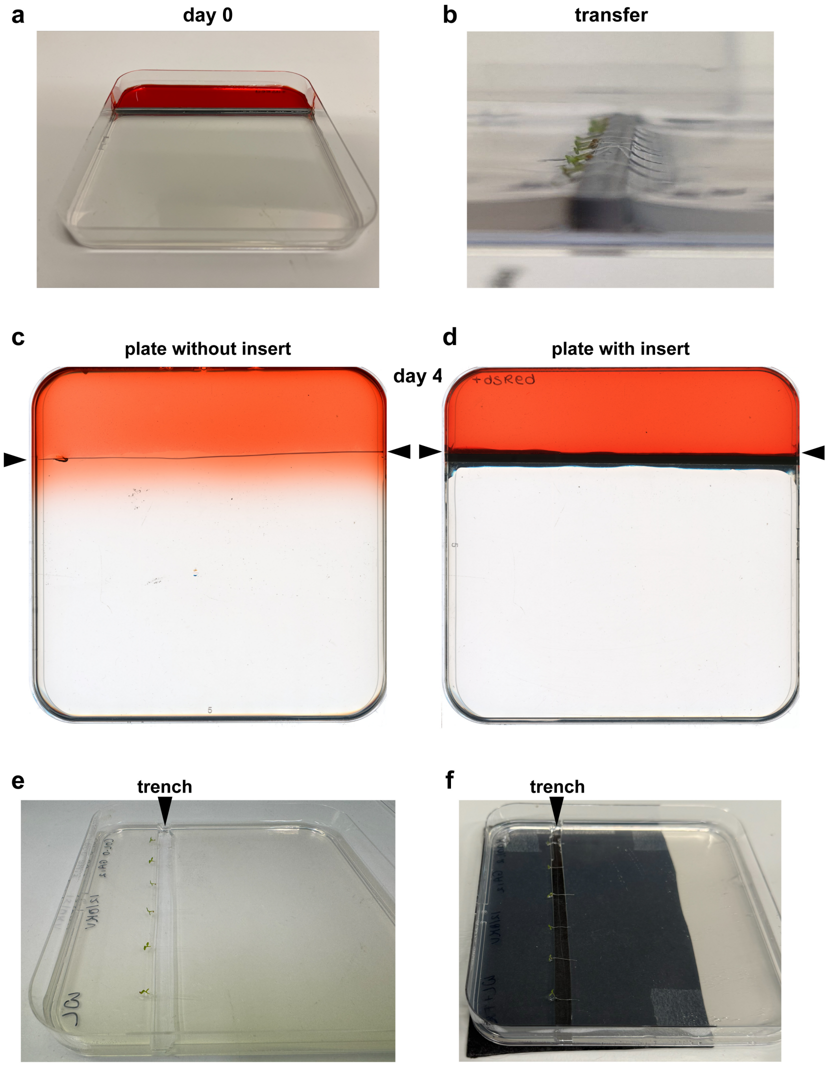

Supplemental Figure S2 | Compartmentalized plates have minimal diffusion after seven days of vertical orientation and seedlings are carefully positioned across the border. Supports Figure 4 (a) Two-compartment plates were constructed gluing with epoxy-resin a plastic insert of 3mm into an off-the-shelf Greiner 12 cm square plate. (b) Close-up image of four-day old seedlings transferred onto the two-compartment plate, with the hypocotyl and shoot part touching the top layer, and the root part touching the bottom layer. (c,d) Comparison of dye diffusion between a plate without border and a plate with. Two agar layers were poured into the plates, a top one containing 0.1% direct red 23, and a bottom one, without any addition. After 4 days of vertical placement, no leakage of the dye had occurred in the plate with border, while a plate without border had clear diffusion of the dye, demonstrating that the two layers can stay sufficiently separated. (e,f) For figure 5, a trench system has been used to separate top and bottom compartments. An agar layer is poured, and then a trench is removed with a scalpel. GA_{12} treatments were then applied through a droplet of 0.1% agarose on the shoot.

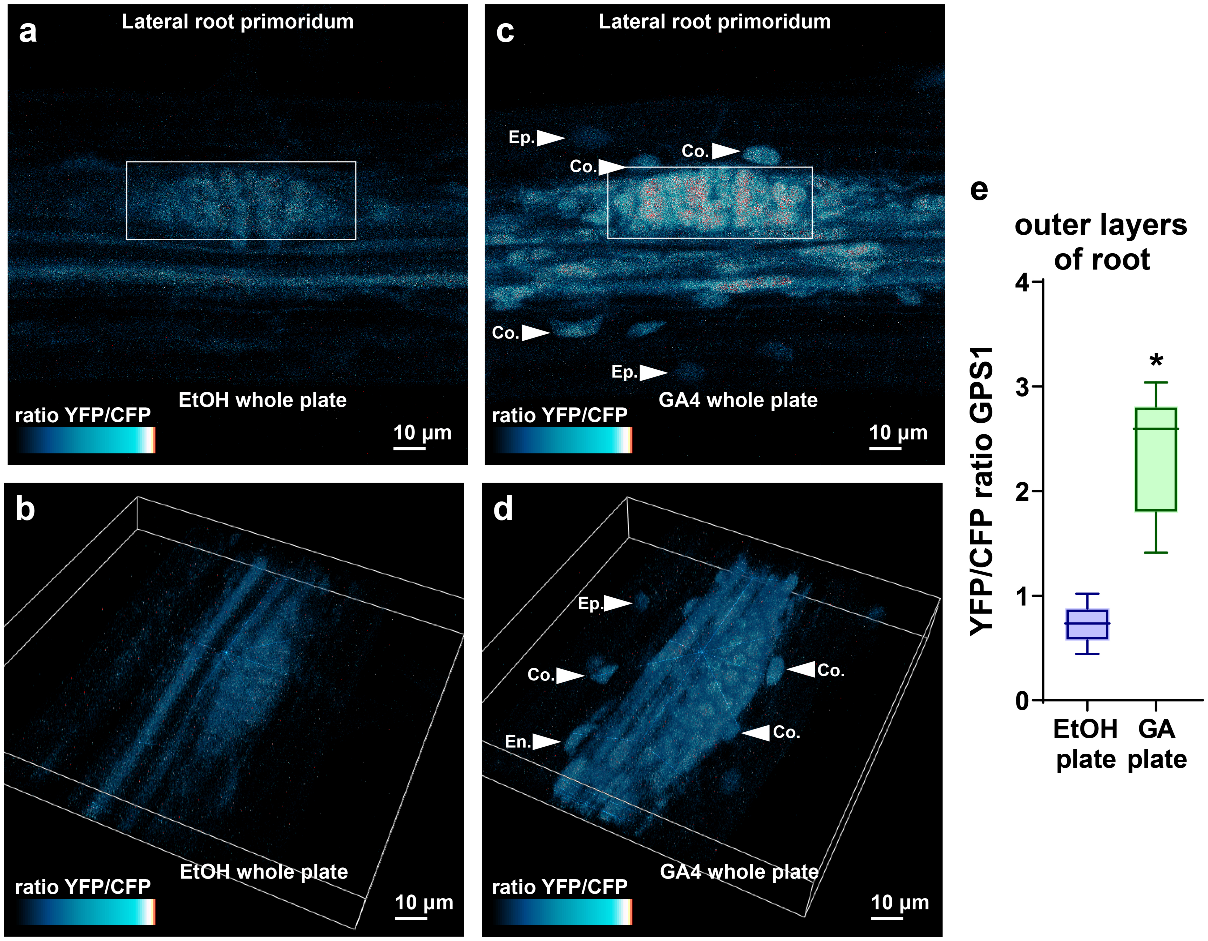

Supplemental Figure S3 | GPS1 ratios in the outer root layers (cortex, epidermis) increase strongly with a whole plate GA4 treatment. Supports Figure 6. (a-d) Representative images of stage 4 lateral root primordia of six day old seedlings from the GPS1 line treated with mock or GA4. Shown is the ratio between YFP and CFP emission of GPS1 (a,c) 2D projection, (b,d) 3D projection, White box emphasizes the LRP, and nuclei of the epidermis (Ep.), cortex (Co.) and endodermis (En.) layers have been highlighted .white arrow denotes cortex and epidermis cell nuclei with an increase in GPS1 ratio. (e) Quantification of images from the whole experiment. *significant difference based on student’s t-test ($p<0.05$), n = 9. Boxplot whiskers show the distribution.

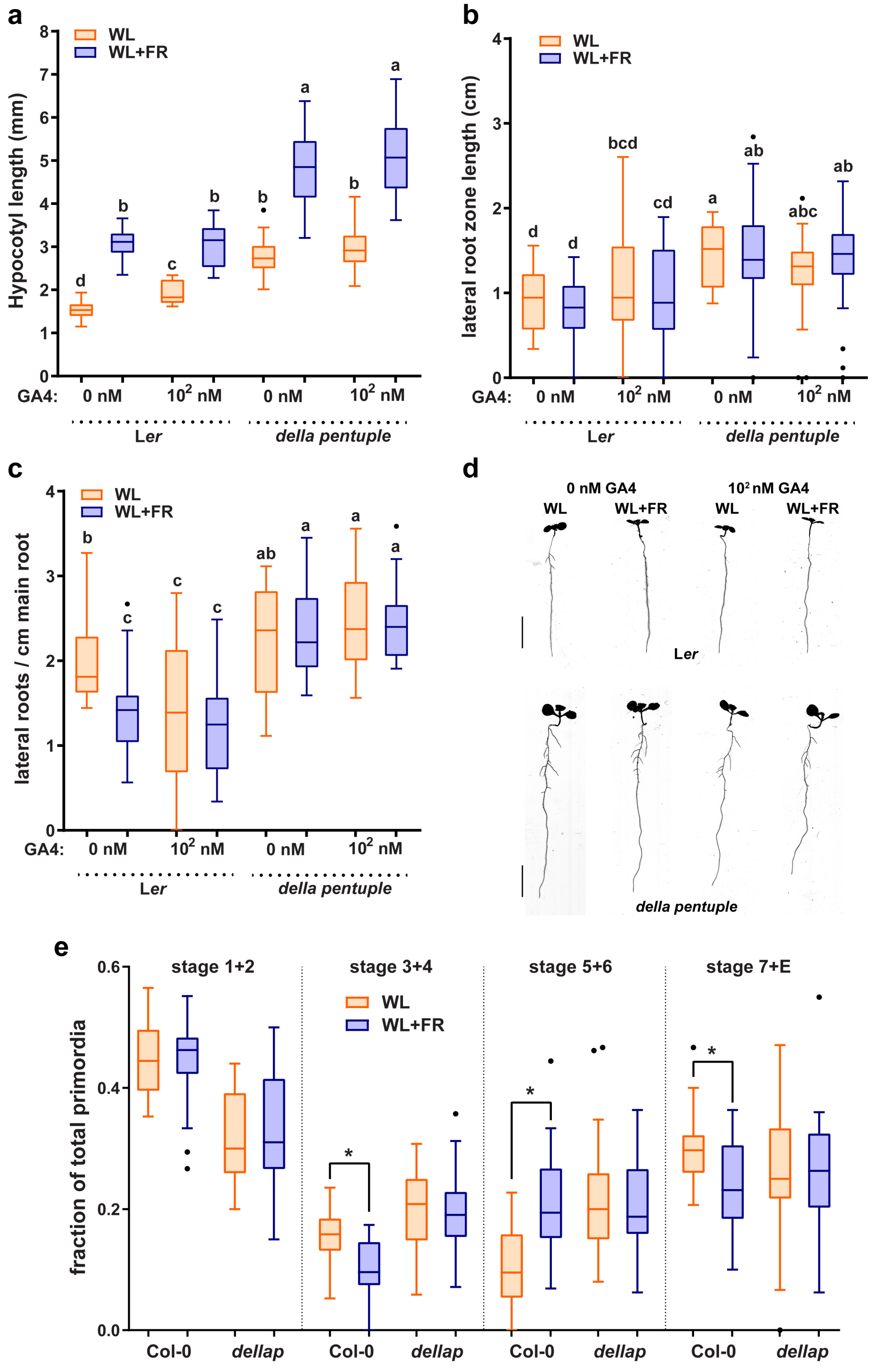

Supplemental Figure S4 | The *della pentuple* mutant has no lateral root development reduction in WL+FR and does not respond to a GA4 treatment. Supports Figure 7. Seedlings of L*er* and *della pentuple* were grown for 9 days according to the schedule on Fig. S1a. Scans of 9d old seedlings treated with EtOH 1:10000 in the medium or 10^2 nM GA4 and analyzed on (a) hypocotyl length; (b) lateral root density; (c) lateral root zone length. (d) Representative seedling images of the experiment in (a-c). scale bar = 1 cm. Means were statistically significant based on a 2-way ANOVA; letters denote significant difference between treatments based on a post-hoc tukey test ($p<0.05$, $n > 20 < 30$). Boxplot whiskers show the distribution.

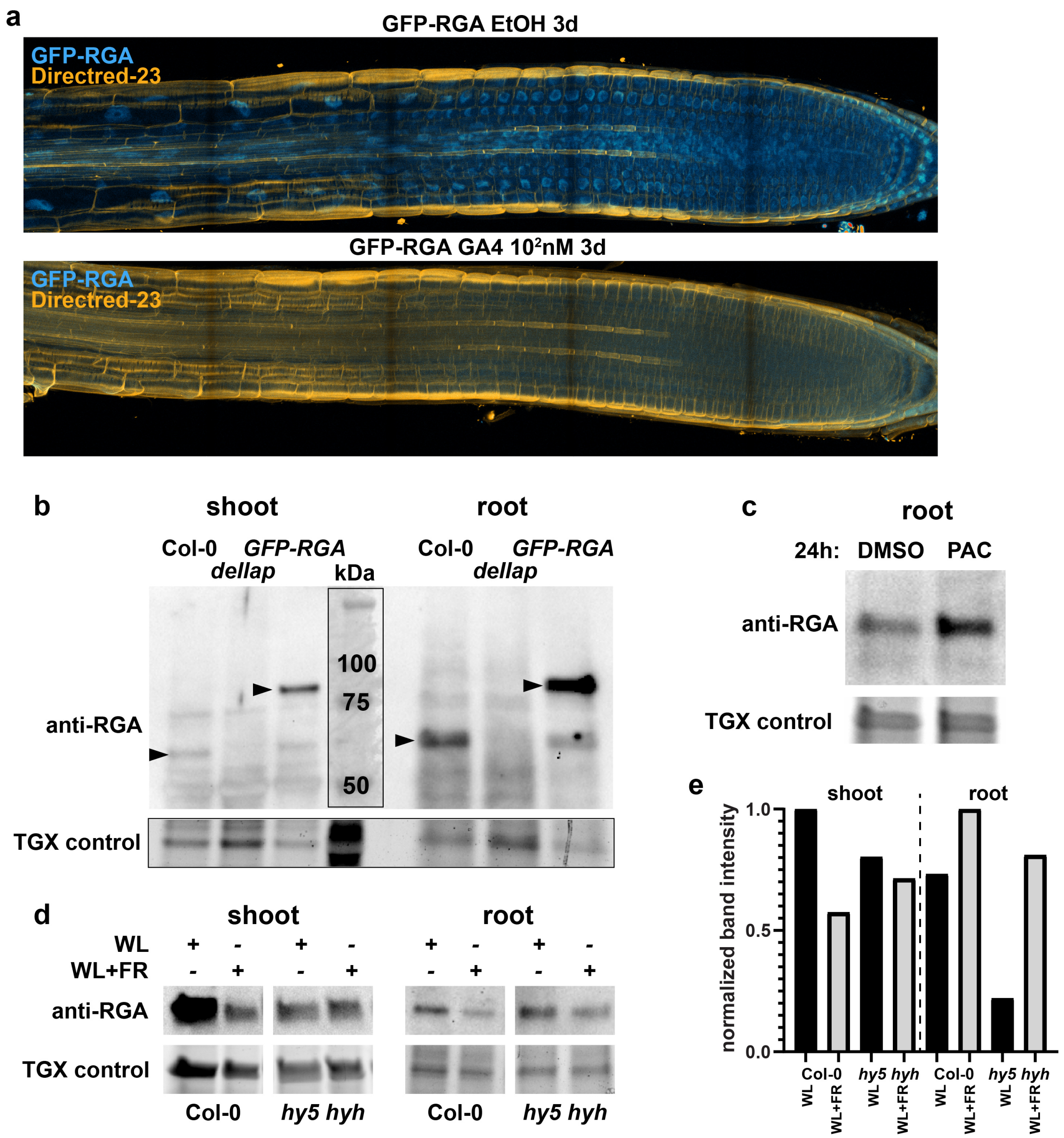

Supplemental Figure S5 | WL+FR regulates RGA stability in the root and shoot and is gated by the *hy5 hyh* mutations. Supports Figure 8. (a) Microscopy images of 7 day old seedling roots of *rga pRGA:GFP-RGA*, treated with mock EtOH or with 10^2 nM of GA4. Multiple images were stitched together to show the entire sample. (b) Western blot experiment using 5 day old seedling shoots and roots of Col-0, *della pentuple* and of *rga pRGA:GFP-RGA,* demonstrating the specificity of the RGA antibody. (c) Western blot experiment using 5 day old seedling roots of Col-0 treated for 24 hours with mock (DMSO) or 1 µM paclobutrazol. (d) Western blot experiment using 5 day old seedling shoots and roots of Col-0 and *hy5 hyh,* grown either in WL, or in WL+FR (for 24 hours). (e) Quantification of bands in blot (d).

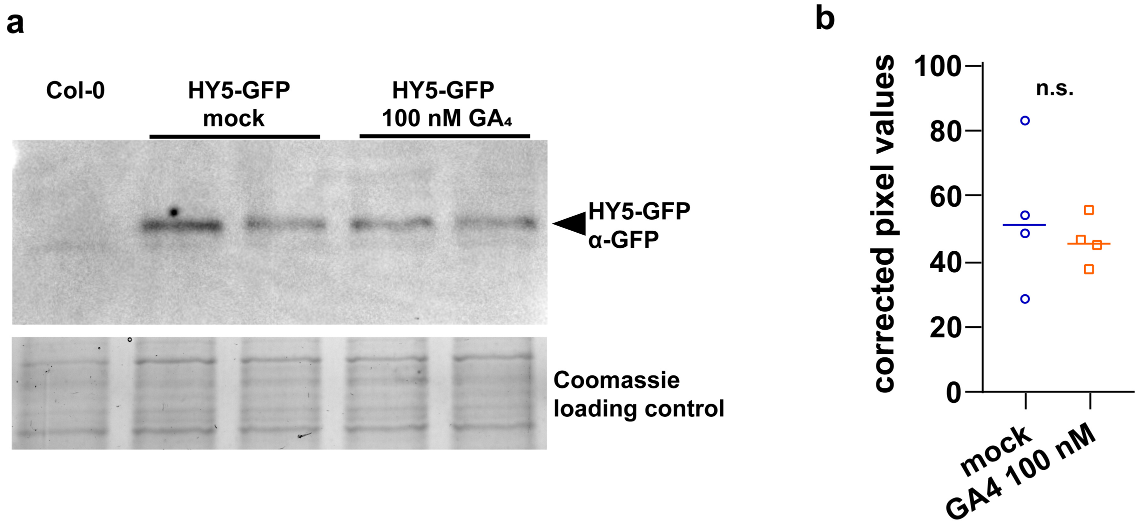

Supplemental Figure 6 | Western blot on HY5-GFP root samples shows no significant difference. Supports Figure 8. (a) Western blot of 6 day-old seedling root samples of Col-0 (control) and *hy5-2 pHY5:HY5-GFP* treated with either mock (EtOH 1:10000) or 100 nM GA_4. Shown are two biological replicates. (b) Quantification of western blot on (a) and one additional, resulting in 4 biological replicates. Difference of mean was not significant, based on a student's t-test ($p>0.05$). Bands were normalized against background signal and the Coomassie loading control.

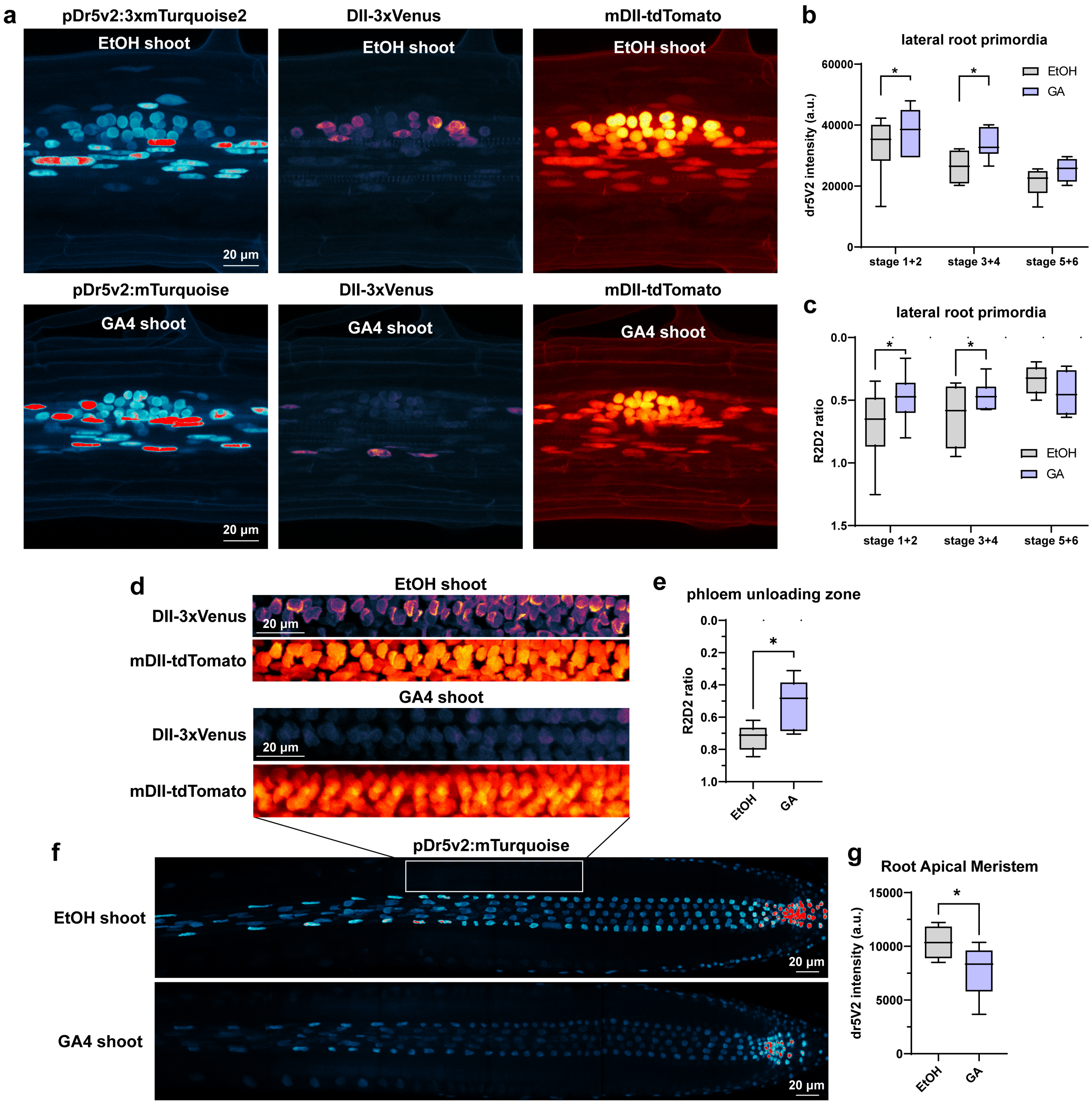

Supplemental Figure S7 | GA4 shoot treatment leads to changes in auxin levels and signaling in root tissues. Supports Figure 8. Confocal microscopy experiment, comparable to Fig. 5, with the C3PO line, containing the R2D2 auxin level sensor and the Dr5v2 auxin signaling sensor. (a,b,c) C3PO line was either treated for two days with shoot GA4, or with mock EtOH, representative images of a stage 4 lateral root primordium (a) and quantifications of the dataset (b,c). (d) Representative images of R2D2 in the phloem unloading zone (for reference, see white box in (f)), with corresponding quantifications (e). (f) Representative images of *pDR5v2:mTurquoise* in the main root tip, white box denotes the phloem unloading zone, with quantification of dataset in (g). Multiple images were stitched together to show the entire sample. (b,c) Means were statistically significant based on a 2-way ANOVA. All graphs: * significant difference based on student's t-test ($p<0.05$, $n > 10 < 20$). Boxplot whiskers show the distribution.

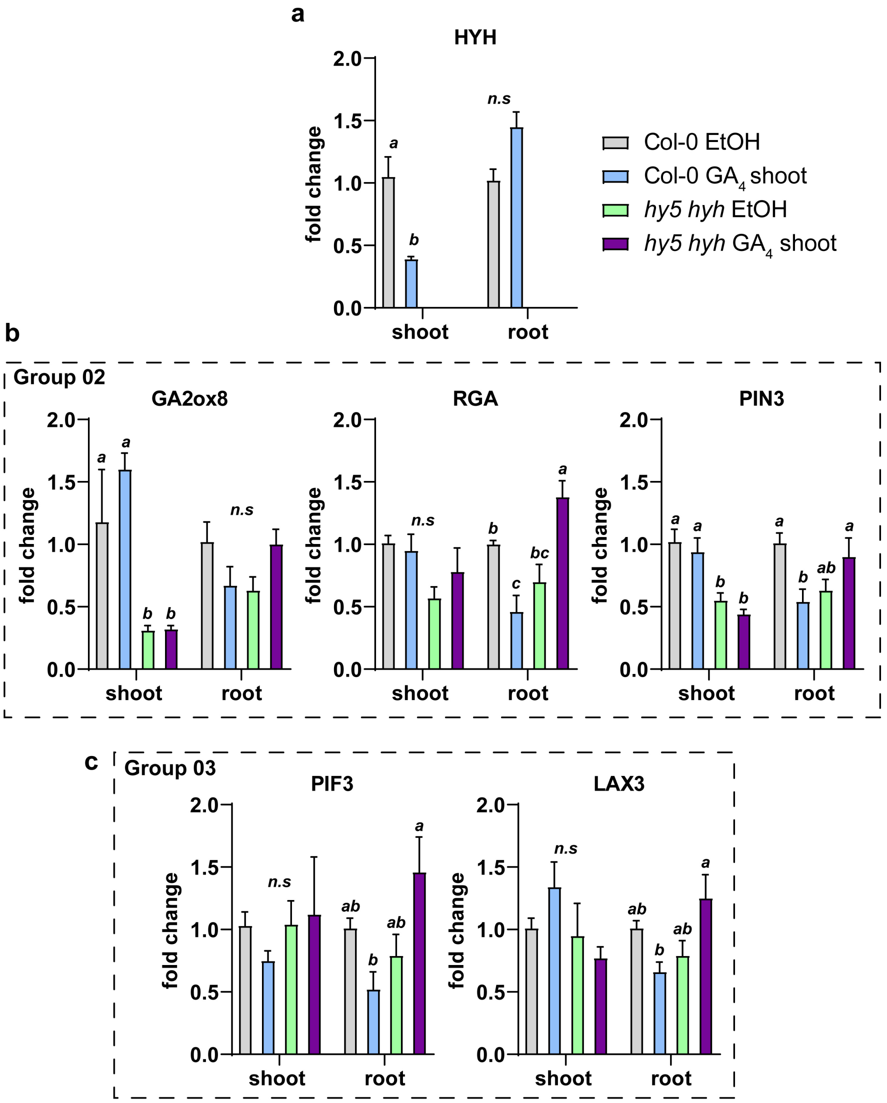

Supplemental Figure S8 | qPCR experiment with shoot GA4 treatment and *hy5 hyh* mutant, supplementary to Figure 8. qRT-PCR analysis of shoot and root samples of 5 day old seedlings (Col-0 and *hy5 hyh*), transferred to mock or GA4-shoot 24hrs before harvesting. (a) *HYH* expression values (not detectable in *hy5 hyh* mutant). (b) Additional cluster 02 genes, (c) Cluster 03 genes. Expression values were analyzed according to the $\Delta\Delta^{CT}$ method and normalized against the Col-0 mock sample for both shoot and root respectively. Four biological replicates per treatment, Error bars denote the standard error of the mean. Statistical significance was determined by 2-way ANOVA with a Newman-Keuls post-hoc test (p>0.05).

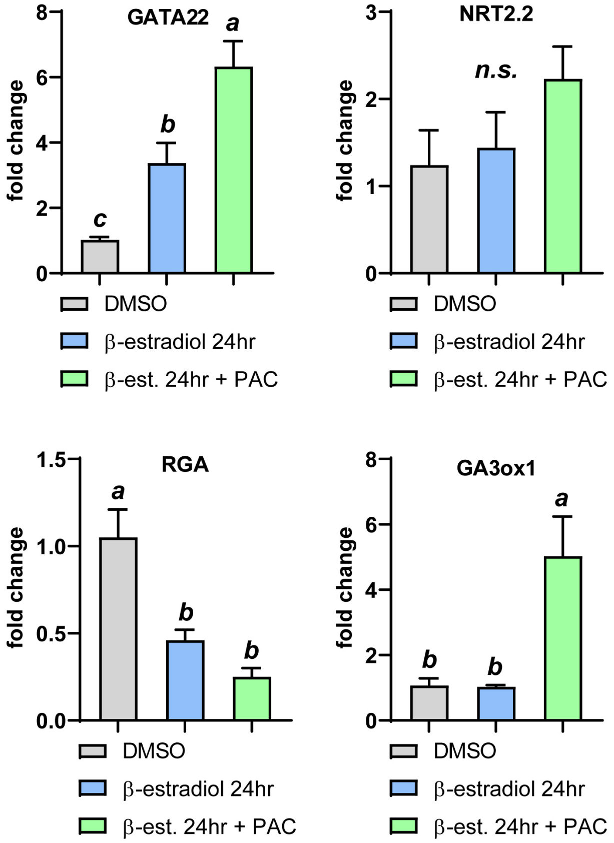

Supplemental Figure S9 | Additional qPCR data to Figure 9 qRT-PCR analysis of root samples of 5 day old seedlings (Col-0 *pGATA23-XVE:HIS-HY5-YFP*), induced on the root by beta-estradiol, mock induced and treated with paclobutrazol (PAC) 24hrs before harvesting. Expression values were analyzed according to the $\Delta\Delta^{CT}$ method and normalized against the Col-0 mock sample for both shoot and root respectively. Four biological replicates per treatment, Error bars denote the standard error of the mean. Statistical significance was determined by 1-way ANOVA with a Newman-Keuls post-hoc test (p>0.05).

Supplementary Table 1. Primers used in this study.

NAME	SEQUENCE	GENE	DESCRIPTION
hy5-215 FW	ATCAAGCAGCGAGAGGTCAT	AT5G11260	*hy5-215 genotyping (sequencing)*
hy5-215 rev	TTCAGCCGCTTGTTCTCTTT	AT5G11260	*hy5-215 genotyping (sequencing)*
SALK LB	CGTCAAGCTCTAAATCGGGG	left border primer for SALK insertion lines	
HY5 N679707 LP	TTCACTCTCGATATCCGTTCG	AT5G11260	*hy5 SALK_056405C genotyping (+SALK LB)*
HY5 N679707 RP	ATGCGAGTGAATGACCATTTC	AT5G11260	*hy5 SALK_056405C genotyping (+SALK LB)*
HYH wiscdslox RP	ACTCGCATAAGAACATGTGGG	AT3G17609	*WiscDsLox253D10 genotyping*
HYH wiscdslox LP	ACCCACACGCTCTGTGAATAC	AT3G17609	*WiscDsLox253D10 genotyping*
p745 BP	AACGTCCGCAATGTGTTATTAAGTTGTC	AT3G17609	*WiscDsLox left border primer*
NRT2:1 Fw	TGGAGAAAGCGGGAGAAGTT	AT1G08090	*NRT2:1* qPCR primer fw
NRT2:1 Rev	GCTCAACTCAACTCCCATGG	AT1G08090	*NRT2:1* qPCR primer rev
HY5 CDSmiddle FW	TGAGCGAGTTGGAAAACAGA	AT5G11260	*HY5* qPCR primer fw
HY5 RV Zheng, Zhang	AAGGCTTGCATCAGCATTAG	AT5G11260	*HY5* qPCR primer rev
GA4_FWQ	CCCATTCACCTCCCACACTC	AT1G15550	*GA3ox1* qPCR primer fw
GA4_RVQ	GCAGCGGAGAAGAGGAGATC	AT1G15550	*GA3ox1* qPCR primer rev
ARF19 qPCR FW	AGGCTCACAATGGCGTAATC	AT1G19220	*ARF19* qPCR primer fw
ARF19 qPCR rev	AAGGAGTTATGACGGGTTCG	AT1G19220	*ARF19* qPCR primer rev
IAA2_qPCR_fw	GCGTCTATTTGAGGAAACTCG	AT3G23030	*IAA2* qPCR primer fw
IAA2_qPCR_rv	AGGAGCTCCGTCCATACTCA	AT3G23030	*IAA2* qPCR primer rev
BBX21 qPCR fw	GGATCCACAAGCACGATTTC	AT1G75540	*BBX21* qPCR primer fw
BBX21 qPCR rev	CACTCCATCATCATCACCACTC	AT1G75540	*BBX21* qPCR primer rev
IDA_qPCR_FW_02	GCGGCGAGTAGTTCTTGTGT	AT1G68765	*IDA* qPCR primer fw
IDA_qPCR_REV_02	GAATGGGAACGCCTTTAGGT	AT1G68765	*IDA* qPCR primer rev
SHY2 fw	GGTGATTGGATGCTCATTGG	AT1G04240	*SHY2* qPCR primer fw

SHY2 rev	GCCTAAACCTTTGGCTTCTG	AT1G04240	*SHY2* qPCR primer rev
HAE_qPCR_fw	TGGGGCTAAAGTTGCTGACT	AT4G28490	*HAESA* qPCR primer fw
HAE_qPCR_rv	TTTCATTCACCCGAAGTGTG	AT4G28490	*HAESA* qPCR primer rev
GA2OX6_qPCR_fw	CCCATCTGACCCTACATGCT	AT1G02400	*GA2ox6* qPCR primer fw
GA2OX6_qPCR_rv	GTACATCGCCGACATACGTG	AT1G02400	*GA2ox6* qPCR primer rev
GA2ox8-ChIP-qPCR-HY5-Fw2	ACCCTTTAGCTTCTCAGTCACAG	AT4G21200	*GA2ox8* (ChIP)qPCR primer fw
GA2ox8-ChIP-qPCR-HY5-Rev2	TCTCGCAATCGCTTCCTTAC	AT4G21200	*GA2ox8* (ChIP)qPCR primer rev
RGA FW	GAACCAAGCGATTCTCGAAG	AT2G01570	*RGA* qPCR primer fw
RGA REV	TAACCGGAAAGTTGGAGGAC	AT2G01570	*RGA* qPCR primer rev
PIN3_F	ATCTTCTCACCCGACCAATG	AT1G70940	*PIN3* qPCR primer
PIN3_R	GATGCTCCACTCGAGGCTAC	AT1G70940	*PIN3* qPCR primer
LAX3fw	ACTGTCTACATCATCCCCGC	AT1G77690	*LAX3* qPCR primer
LAX3rev	ACCCGAACCCAACTACGAAT	AT1G77690	*LAX3* qPCR primer
HYH qPCR_KO FW	GCTTGACCAGACTCAAAATGG	AT3G17609	*HYH* qPCR primer fw
HYH qPCR KO rev	TGCTTGTTGCGCTGATACTC	AT3G17609	*HYH* qPCR primer rev
Pif3_F	GAATCTGCTCAAGACAGGAAC	AT1G09530	*PIF3* qPCR primer fw
Pif3_R	CTCGTTGACAGTAACAGGAGAC	AT1G09530	*PIF3* qPCR primer rev
PIN3_F	ATCTTCTCACCCGACCAATG	AT1G70940	*PIN3* qPCR primer
PIN3_R	GATGCTCCACTCGAGGCTAC	AT1G70940	*PIN3* qPCR primer
LAX3fw	ACTGTCTACATCATCCCCGC	AT1G77690	*LAX3* qPCR primer
LAX3rev	ACCCGAACCCAACTACGAAT	AT1G77690	*LAX3* qPCR primer
pFBP fw	TCTTCTTGTACCCGGCTGACA	AT1G43670	*FBP* ChIP-qPCR primer fw
pFBP rev	CTCCGGCTTGCTCCATCAAA	AT1G43670	*FBP* ChIP-qPCR primer rev
OCP3 HY5 binding site fw	GCAGCAGTGAGAAAAATAATCG	ATG5G11270	*HY5* ChIP-qPCR primer fw

OCP3 HY5 binding site rev	AACGACACGCTTTGCTACAC	ATG5G11270	*HY5* ChIP-qPCR primer rev
GA2OX6-ChIP-qPCR-HY5-Fw	TTAAATCGGACGGCTAATGG	AT1G02400	*GA2ox6* ChIP-qPCR primer fw
GA2OX6-ChIP-qPCR-HY5-Rev	TCACAAGGAAGTGGGAAAGG	AT1G02400	GA2ox6 ChIP-qPCR primer rev
NRT2.1-ChIP-qPCR-HY5-Fw	CCCAACTTGTTGGAAATTTGAC	AT1G08090	*NRT2:1* ChIP-qPCR primer fw
NRT2.1-ChIP-qPCR-HY5-Rev	TCCGTACTCTTGTAGCGATGG	AT1G08090	*NRT2:1* ChIP-qPCR primer rev
LAX3-ChIP-qPCR-HY5-Fw	CAGCTCACGAGTTGTCATGG	AT1G77690	*LAX3* ChIP-qPCR primer fw
LAX3-ChIP-qPCR-HY5-Rev	TCGCCGAAAACAGTAACAGTC	AT1G77690	*LAX3* ChIP-qPCR primer rev
SHY2-ChIP-qPCR-HY5-Fw	TTGCCCTACAGAGCAGTAGAAG	AT1G04240	*SHY2* ChIP-qPCR primer fw
SHY2-ChIP-qPCR-HY5-Rev	CGATTCTGTGATCAGATGTGG	AT1G04240	*SHY2* ChIP-qPCR primer rev
HAE-ChIP-qPCR-HY5-Fw	CAAGCCAAACTGGGCTTATC	AT4G28490	*HAESA* ChIP-qPCR primer fw
HAE-ChIP-qPCR-HY5-Rev	GCATGAAGCTTGAGAGATCG	AT4G28490	*HAESA* ChIP-qPCR primer rev
GATA22 FW	aacaacgattgcgtgattagg	AT4G26150	qPCR primer fw
GATA22 Rev	gccttcctttgccttattcc	AT4G26150	qPCR primer rev
NRT2.2 qPCR FW	CGCCGTTACAAATTACAGGAC	AT1G08100	*NRT2:2* qPCR primer fw
NRT2.2 qPCR rev	ATAATCCCCGCTGTGTGAAG	AT1G08100	*NRT2:2* qPCR primer rev
rga1-3_rev	GCGTTTTGTTGGAATGTTCG	AT4G02780	genotyping
ga1-3_fwd	CGTTTGGATTGGCAAGACTC	AT4G02780	genotyping
ga1-fwd	CCTAACGGACGGGGAAATTA	AT5G25900	genotyping
ga1_rev	TACCGGTTACTGCTTGGTTT	AT5G25900	genotyping
rga-t2 Fw	GCCGGAGCTATGAGAAAAGTGG	AT2G01570	genotyping
rga-t2 Re	AAGAATTTTAAACAAGTGAACG	AT2G01570	genotyping
rgl1-1 Fw	AAAGCTAGCTCGAAACCCCAA	AT1G66350	genotyping
rgl1-1 Re	CCACAGAGCGCGTAGAGGATAAC	AT1G66350	genotyping
rgl2-1 Fw	GCTGGTGAAACGCGTGGGAACA	AT3G03450	genotyping
rgl2-1 Re	ACGCCGAGGTTGTGATGAGTG	AT3G03450	genotyping
RGL3-F2	GGCGATTCTAGAAGCTGTT	AT5G17490	genotyping

RGL3-R1	AGTCGAACAAGCTCGAGTAA	AT5G17490	genotyping
GAI N6	TTATTAGAAGTGGTAGTGGAGT	AT1G14920	genotyping
GAI R1	CCTCCAATGTCACATGCAAA	AT1G14920	genotyping

Supplementary Table 2. Gene accession numbers

gene name	gene accession number
ARF19	AT1G19220
BBX21	AT1G75540
GA1	AT4G02780
GA2OX6	AT1G02400
GA2OX8	AT4G21200
GA3OX1	AT1G15550
GATA22	AT4G26150
HAE	AT4G28490
HY5	AT5G11260
HYH	AT3G17609
IAA2	AT3G23030
IDA	AT1G68765
KAO1	AT1G05160
KAO2	AT2G32440
LAX3	AT1G77690
NRT2.1	AT1G08090
NRT2.2	AT1G08100
PIF3	AT1G09530
PIN3	AT1G70940
RGA	AT2G01570
SHY2	AT1G04240
